# BFLF1 gene polymorphisms are associated with increased mortality in patients with EBV-associated diseases

**DOI:** 10.1186/s41065-026-00708-4

**Published:** 2026-07-14

**Authors:** Xurong Li, Jing Wu, Shenglei Yu, Zhe Zhou, Wenhong Zhang, Lingyun Shao, Ning Jiang, Qingluan Yang

**Affiliations:** Department of Infectious Diseases, Shanghai Key Laboratory of Infectious Diseases and Biosafety Emergency Response, Huashan Hospital, National Medical Center for Infectious Diseases, Fudan University, 12 Wulumuqi Zhong Road, Shanghai, China

**Keywords:** Epstein-Barr virus, Infectious mononucleosis, Chronic active EB virus infection, NK/T lymphoma, Hemophagocytic lymphohistiocystosis, Genomic variations

## Abstract

**Objective:**

Epstein-Barr virus (EBV) -associated diseases comprise a spectrum of conditions, including infectious mononucleosis (IM) and chronic active EBV infections (CAEBV), as well as malignant lymphomas. The study aimed to compare genomic variations of Type 1 EBV across different subtypes of EBV-associated diseases to investigate the impact of EBV variants on disease progression and clinical outcomes.

**Methods:**

A total of 36 patients with EBV-associated diseases were enrolled. Targeted EBV genome sequencing on EBV genomic was performed with peripheral blood DNA samples (400µL) using Illumina sequencing technology. Following quality control with FastQC, Fastp, and TriTrimmomatic, 27 samples comprising 2 IM, 9 CAEBV and 16 NK/T-cell lymphoma cases meeting the critera of minimum effective coverage > 10 and coverage > 80% were retained. Single nucleotide polymorphisms (SNP) annotations were generated into VCF files, and variant profiles were visualized using heatmaps. Variant rates were compared using Fisher’s exact test, based on disease subtype, presence of hemophagocytic lymphohistiocystosis (HLH), and clinical outcomes. Odds ratios (ORs) were calculated using the Haldane-Anscombe correction.

**Results:**

Compared with the reference stain B95.8/Raji, a total of 2182 variants were identified in 27 samples. The variants located in coding region included 523 missense variants, 1 insertion, and 2 deletion, all of which only involved single amino acid changes in the encoded proteins. Although a large number of variants were detected, no statistically significant differences were observed among the IM, CAEBV and lymphoma groups, or between patients with and without HLH. However, three variants in BFLF1 were significantly more frequent in deceased patients compared to survivors, which detailedly refers to Ile35Thr, Asp44Glu and Val246Ile [*P* = 0.0267, OR = 15.00, 95%CI= (0.7436,301.5)].

**Conclusion:**

Single amino acid changes in EBV proteins had no significant effect on the progression status of EBV-associated diseases, but the variants in BFLF1 were associated with an increased risk of mortality.

**Supplementary Information:**

The online version contains supplementary material available at https://doi.org/10.1186/s41065-026-00708-4.

Epstein-Barr virus (EBV), first identified by Epstein and Barr in 1964, is a widespread human herpesvirus. Its genome is a linear double-stranded DNA (dsDNA) approximately 172 kb in length, encoding 85 genes and separated by four internal repeat sequences (IRs) and terminal repeat sequences (TRs) at both ends [1]. Upon binding to complement receptors (CD21) through its membrane glycoproteins and entering target cells, EBV DNA remains in the cytoplasm, enabling long-term latency. The life cycle of EBV consists of two phases: latent infection and lytic replication. During latency, it mainly expresses EBV nuclear antigen (EBNA) and membrane proteins (LMP) while in the lytic phase, early antigen (EA) and capsid antigen (CA) are produced to utilize in viral assembly and cell lysis [2].

EBV primarily infects epithelial cells and B cells. Latent EBV infection may induce immortalization of target lymphocytes, which is associated with lymphoproliferative disorders. The main types of Epstein-Barr virus (EBV)-associated diseases include: infectious mononucleosis (IM), chronic active EBV infections (CAEBV), and carcinoma (lymphoma such as B-cell Burkitt lymphoma, Hodgkin’s lymphoma, extranasal NK/T-type lymphoma, aggressive NK cell leukemia, and non-specific peripheral T lymphoma and epithelial malignancies such as nasopharyngeal carcinoma and EBV-associated gastric adenocarcinoma) [3]. In some cases, acute primary infection triggers IM, characterized as benign lymphoid hyperplasia in B cells. The course usually lasts about one month, with most patients achieving complete remission. Only a small number of patients experience persistent EBV infection in hematopoietic stem or lymphoid progenitor cells in the bone marrow that later differentiate into T/NK cells. The infected lymphocytes subsequently undergo clonal expansion, immune evasion, and progressive accumulation of genetic and epigenetic alterations, giving rise to systemic CAEBV disease, with a poor prognosis of lymphoma progression or other severe complications [4]. Hemophagocytic lymphohistiocystosis (HLH) is one of the common complications characterized by excessive inflammatory responses. the feature of which involves abnormal proliferation of activated lymphocytes and histiocytes, triggering cytokine storm that induces systemic inflammation and often leading to life-threatening conditions [5].

Accumulating evidence has established that the genetic diversity and specific polymorphic lineaging of the EBV genome are heavily coupled with geographic distributions and tumor susceptibilities. However, while previous genomic sequencing efforts have primarily focused on epithelial malignancies, gene polymorphism governing the clinical transition across different lymphocyte-infiltrating diseases especially CAEBV, this rare pre-malignant lymphoproliferative disease, remains poorly characterized. Currently, robust viral genetic biomarkers capable of predicting this severe phenotypic evolution and fatal prognosis are critically lacking.

To investigate whether the EBV strain itself contributes to different disease manifestations and clinical outcomes, we developed an EBV-specific probe library and utilized Illumina technology to generate corresponding EBV DNA sequences and then compared them with the reference sequence B95.8/Raji (NC007605). This comparative analysis aimed to investigate how nucleotide polymorphisms in EBV DNA sequences influence disease progression and clinical outcomes.

## Methods

### Patients

Our study enrolled 36 patients diagnosed with EBV-associated disease at Huashan Hospital, Fudan University. The cohort comprised 5 cases of IM, 10 cases of CAEBV, and 21 cases of NK/T lymphoma. All participants were adults (aged ≥ 18 years) and excluded individuals with diabetes, autoimmune diseases, or other chronic infections (including HBV/HCV, CMV, HIV, and tuberculosis). Informed consent was obtained from all subjects and the study protocol was approved by the Ethics Committee of Huashan Hospital, Fudan University.

Diagnostic Criteria for IM [6]: The patient must meet any three of the following clinical manifestations and one of laboratory evidences of primary EBV infection: (1) Clinical manifestations: ① fever; ② pharyngitis; ③ enlarged cervical lymph nodes; ④ hepatosplenomegaly; ⑤ rash; ⑥ eyelid edema; (2) Laboratory findings: ① positive anti-EBV-capsid antigen (CA)-IgM and anti-EBV-CA-IgG with negative anti-EBV-nuclear antigen (NA)-IgG; ② positive single anti-EBV-CA-IgG antibody with low-affinity binding to EBV-CA-IgG; (3) Exclusion of acute infection caused by other pathogens.

Diagnostic criteria for CAEBV [7]: (1) Persistent or recurrent infectious mononucleosis-like symptoms lasting over three months; (2) Increased EBV genomic load in peripheral blood and/or affected tissues (elevated EBV DNA or positive EBV EER); (3) EBV infection affecting T cells, NK cells, or multiple cell lineages (two or more types including T, B, or NK); (4) Exclusion of other chronic diseases that may cause similar symptoms. EBV-associated NK/T lymphoma can be diagnosed according to WHO-HAEM5 and increased EBV genome in peripheral blood and/or affected tissues (elevated EBV DNA or EBER positive) [8]. EBV-associated HLH can be diagnosed according HLH-2024 [9]: Increased EBV genomic load in peripheral blood and/or affected tissues (elevated EBV DNA or positive EBV DNA test).

### EBV genomic sequencing

Genomic DNA was isolated from 400 µL of peripheral blood. DNA mass was quantified fluorometrically using Qubit (Thermo Fisher Scientific). The EBV DNA load was determined by absolute quantification qPCR targeting the BKRF1 gene (encoding EBNA1) using primers GCCGGTGTGTTCGTATATGG/CAAAACCTCAGCAAATATATGAG. To ensure the ability to capture diverse EBV strains, the capture probes were designed with 4x coverage tiling approach. Typically, 120-bp probes were arranged with a step size of approximately 30 bp, ensuring high coverage depth for uniform hybridization and comprehensive variant capture. The probe design was based on a multiple sequence alignment (MSA) of isolates from different sub-strains of type I EBV genomic sequences. EBV reference genome (GenBank NC_007605) was used as the backbone for target sequence. Then, degenerate or parallel probes were synthesized to cover known single nucleotide polymorphisms and insertion/deletion sites. Probes are aligned against the host (human) reference genome using BLASTN, and sequences with high homology to the human genome are removed to reduce off-target capture. Threshold cycle (CT) values were compared to a standard curve generated from a linearized plasmid containing a 598-bp target sequence amplified from the EBV genome (primers: AGGCCATTTTTCCACCCTGT/CGTCAAAGCTGCACACAGTC) to calculate EBV genome copies per microliter. Samples exceeding 5.0 × 10³ copies/µg DNA were selected for sequencing.

Genomic DNA (1 µg) was fragmented to an average size of ~ 250 bp using the Covaris ME220 System (Covaris, Woburn, MA) according to the manufacturer’s recommendations. Subsequent library preparation steps—including end repair, 3′-dA tailing, Illumina-compatible adapter ligation, and post-ligation cleanup—were performed according to the SureSelectXT Illumina Paired-End Sequencing Library protocol (Agilent Technologies). Target enrichment was performed using the SureSelectXT Target Enrichment Kit (Agilent Technologies, USA) with hybridization at 65 °C, strictly following the manufacturer’s protocol. Post-hybridization capture cleanup and enrichment PCR were conducted following the kit instructions. Final enriched libraries were quantified using the Kapa Library Quantification Kit (Kapa Biosystems, Wilmington, MA) on an ABI 9700HT Fast Real-Time PCR system (Thermo Fisher Scientific) and sequenced on the Illumina NovaSeq platform (2 × 150 bp paired-end reads).

### Quality control of raw data

The 36 FastQC files generated by the platform were processed through FastP and Trimmomatic in Java programs, with results shown in supplementary Fig. 1. We removed the adapter sequences and low-quality sequences with average Phred score less than 20 at read ends, and also checked the allele frequency of each polymorphic site, which was higher than 95%. It means only one dominant EBV strain existing in a patient. We observed that Per Base Sequence Content remained abnormal after processing by both FastP and Trimmomatic, indicating that the issue might not stem from low-quality sequences or adapter contamination, but rather from library construction or probe bias. Given the similar results from FastP and Trimmomatic, we selected FastP as the final fliter. We defined effective coverage as DP > 10 and valid samples as coverage > 80%, ultimately eliminating 9 invalid samples, with results shown in supplementary Table 1. We generated BAM files by alignment strategy to map the clean NGS reads to EBV reference genomic sequencing with Bowtie2 algorithm. The remaining 27 valid samples were further analyzed, including 2 cases of IM, 9 cases of CAEBV, and 16 cases of NK/T lymphoma.

**Fig. 1:**
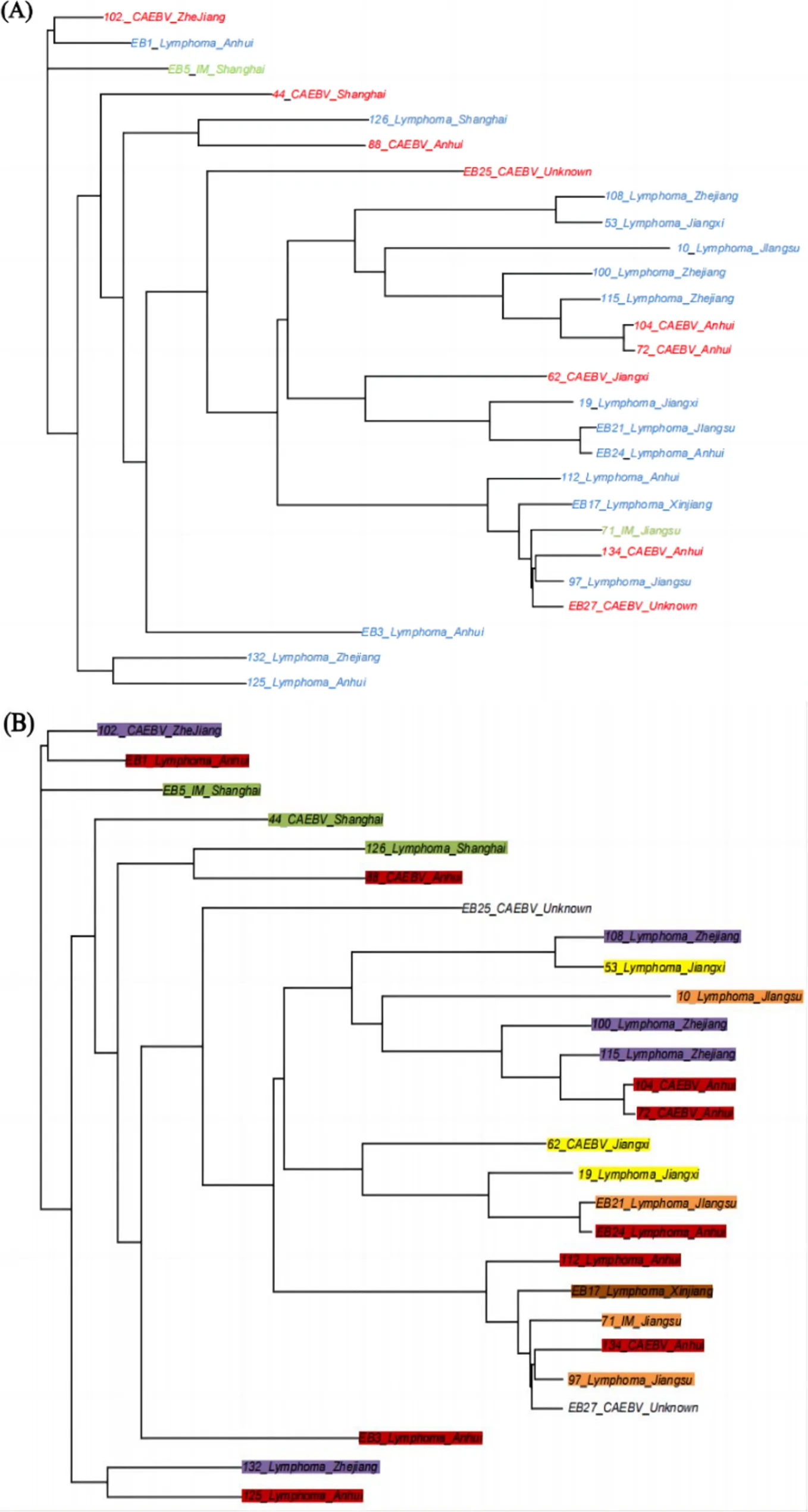
Phylogenetic map of patients with different EBV-associated diseases (**A**) (red-CAEBV, blue- Lymphoma and green-IM) and from different regions (**B**) (red-Anhui, orange-Jiangsu, yellow-Jiangxi, green-Shanghai, purple-Zhejiang, white-unknown)

### Variant annotation

Using B95.8/Raji (NC007605) as the reference sequence, we performed variant detection on 27 valid samples through VCF analysis, covering single nucleotide polymorphisms (SNPs), insertions, and deletions. As EBV is haploid with only one allele per locus, the VCF annotation directly uses 0 (reference allele) and 1 (variant allele) markers. To minimize false-positive variant calls resulting from alignment ambiguities in highly repetitive sequences, variants located within the major repeat regions of the EBV reference genome (B95-8, NC_007605.1) were strictly excluded from downstream analyses. These excluded regions comprised the Terminal Repeats (TR; coords 170,000–171,823 bp), Internal Repeats 1 (IR1; coords 12,000–35,000 bp) and Internal Repeats 2 (IR2; coords 39,800–41,200 bp). We also utilized bioinformatics tools to generate phylogenetic trees for visualizing genetic variant patterns.

### Structural prediction of mutant protein

We obtained relevant information of BFLF1 protein of B95.8/Raji from the UniProt database, including its amino acid sequence and spatial structure. Based on the amino acid changes caused by DNA variant, we generated the amino acid sequence of the mutant protein. Subsequently, both the wild-type and mutant protein sequences were respectively imported into AlphaFold3 to predict their quaternary structures. Finally, ChimeraX was used to visualize the structures.

### Statistical analysis

The presence of variants at a certain site was expressed by 0/1 and displayed in the form of heat map. The comparison of variant rates between groups was expressed in the form of contingency table, and the statistical comparison was made by Fisher exact probability method. The 95% confidence interval (CI) for the odds ratio (OR) was calculated using the Haldane-Anscombe correction. *P* < 0.05 was considered as statistical difference. GraphPad Prism9.5 was used to complete the analysis.

## Results

### Characteristic of enrolled patients

27 patients whose sequencing data meet the criteria of quality control showed no significant difference in any baseline characteristics except hemoglobin (*P* = 0.037) among IM, CAEBV and NK/T lymphoma. NK/T lymphoma patients had a lower number of hemoglobin than other 2 groups indicating that proliferating lymphoma cells would lead to hematopoietic disorder. Notably, EBV-DNA in whole blood from CAEBV patients was higher than that in patients with IM and lymphoma. This discrepancy may reflect the systemic massive expansion of EBV in infected cells in CAEBV. More detailed baseline characteristics are presented in Table 1.

**Table 1:** Basic information of EBV-LPDs patients

		IM	CAEBV	NK/T Lymphoma
Number		2	9	16
Gender [Male (%)]		1 (50%)	6 (66.7%)	11 (68.8%)
Age [M(Q1,Q3)]		20 (15,25)	30 (23,40)	38 (22,55)
Province [count (%)]				
	Anhui	0	4 (44.4%)	5 (31.3%)
	Jiangsu	1 (50%)	0	4 (25%)
	Jiangxi	0	1 (11.1%)	2 (12.5%)
	Shanghai	1 (50%)	1 (11.1%)	1 (6.25%)
	Xinjiang	0	0	1 (6.25%)
	Zhengjiang	0	1 (11.1%)	3 (18.8%)
	Unknown	0	2 (22.2%)	0
Symptoms^1^ [count (%)]				
	Fever	1 (100%)	7 (100%)	13 (86.7%)
	Pharyngalgia	1 (100%)	0	1 (6.67%)
	Rash	0	1 (14.3%)	5 (33.3%)
	Lymphadenectasis	1 (100%)	2 (28.6%)	5 (33.3%)
	Hepatomegaly	0	0	5 (33.3%)
	Splenomegaly	1 (100%)	6 (85.7%)	12 (80%)
Comorbidty [count(%)]				
	Hemophagocytic lymphohistiocytosis	0	2 (22.2%)	7 (43.8%)
	Diabetes	0	0	1 (6.25%)
	Chronic Nephropathy	0	0	0
	HIV infection	0	0	0
Blood routine^2^ [M(Q1,Q3)]				
	WBC count (x10^9^/L)	11.0 (2.37,19.6)	3.60 (2.09,5.60)	2.67 (1.40,6.72)
	Neutrophil ratio (%)	34.1 (15.53.2)	53.5 (32.4,77.8)	59.5 (52.6,79.1)
	Lymphocyte ratio (%)	24.8 (18,31.6)	36.8 (15.0,48.1)	19.8 (11.5,35.3)
	Platelet (x10^9^/L)	142 (121,163)	150 (80,215)	94 (36,143)
	Hemoglubin (g/L)	143 (120,166)	116 (109,142)	91 (81,119)
EBV DNA^3^ [M(Q1,Q3)]				
	Whole blood (x10^6^ copies/L)	1.03 (0.1,1.95)	47.3 (3.25,93.5)	4.44 (0.1,27.7)
	Plasma (x10^4^ copies/L)	0.044 (0.04,0.048)	1.12 (0.78,38.1)	1.58 (1.11,12.9)
PBMC sorting^4^ [M(Q1,Q3)]				
	CD3 + T cell (%)	78.8 (68.7,88.9)	48.4 (38.2,60.1)	49.5 (30.1,78.0)
	CD4 + T cell (%)	30.2 (7.57,52.8)	25.9 (18.8,38.7)	25.2 (18.6,46.8)
	CD8 + T cell (%)	45.5 (13.7,77.4)	14.8 (9.39,30.4)	18.3 (7.61,25.3)
	CD4/CD8 ratio	1.98 (0.1,3.85)	2.13 (0.62,3.36)	1.80 (1.21,2.69)
	CD19 + B cell (%)	7.30 (0.82,13.8)	8.17 (1.30,14.3)	5.53 (3.98,10.1)
	CD56 + NK cell (%)	13.7 (9.93,17.5)	38.6 (26.1,59.9)	31.3 (11.8,66.3)
Outcome [count (%)]				
	Cure	1 (50%)	0	0
	Improvement	1 (50%)	0	3 (18.8%)
	Stable	0	5 (55.6%)	6 (37.5%)
	Death	0	2 (22.2%)	7 (43.8%)
	Loss of follow up	0	2 (22.2%)	0

^1^ 1, 2 and 1 patients in IM group, CAEBV group and lymphoma group respectively failed to record their symptoms.

^2^ 2 patients in CAEBV group refused to test blood routine and 3 patients in lymphoma group failed to categorize neutrophils and lymphocyte due to their insuffient peripheral blood cells.

^3^ 3 patients in CAEBV group refused to quantify EBV DNA both in whole blood and plasma while 2 patients in lymphoma group lost their results only in whole blood.

^4^ 3 patients respectively in CAEBV group and lymphoma group lost their results

### Phylogenetic map

The 27 patients in the final cohort were from six provinces: Anhui, Jiangsu, Jiangxi, Shanghai, Xinjiang, and Zhejiang, indicating considerable regional diversity. To assess whether disease type and geographic distribution were associated with viral genetic variation, we conducted a phylogenetic analysis to identify the EBV strain sources and their relationship. The results revealed that the submitted samples clustered across multiple evolutionary branches, with no significant pathological and geographical pattern observed among them (Fig. 6).

### Variant between different groups

#### Landscape of variant sites in analyzed samples

The sequences of the 27 analyzed samples were compared with the B95.8/Raji sequence (GenBank accession: NC 007605) from the NCBI database and performed SNP annotation. 2182 single nucleotide variants (SNVs), 12 insertion, and 11 deletion were identified relative to the reference. Among these, only 523 were missense variants, and all identified insertions and deletions within the coding regions were strictly in-frame variations. Specifically, one insertion and two deletions resulted in single-amino-acid length changes (Serine duplication on 4 in BRRF1, Glutamine deletion on 61 in BKRF4, and Threoine deletion on 407 in BGLF1; seen in Table 2), while the remaining indels were located within non-coding regions. Because these indels maintained the triplet reading frames, they modified only individual codons rather than causing broader structural disruptions such as reading frameshifts or premature termination codons.

**Table 2:** Variant site of EBV encoding proteins

Encoding protein	Period	Variant site	Mutation site
BSLF2/BMLF1 (Mta)	Immediate early lytic phase (IE)	70,501 C > G	Gly465Ala
		71,677 C > T	Arg73Gln
		71,687 T > C	Ile70Val
		71,766 C > G	Glu43Asp
		71,780 G > T	Leu39Met
		71,975 C > T	Arg9Gln
BRRF1	Immediate early lytic phase (IE)	92,898 + GTA	Ser4dup
		93,017 G > A	Asp42Asn
		93,098 A > G	Lys69Glu
		93,195 A > C	Asp101Ala
		93,368 A > C	Ile159Leu
		93,599 T > G	Ser236Ala
		93,674 C > T	His261Tyr
		93,792 T > C	Val300Ala
BRRF2	Immediate early lytic phase (IE)	94,111 T > A	Val33Asp
		94,179 G > A	Asp56Asn
		94,254 G > A	Asp81Asn
		94,266 G > A	Ala85Thr
		94,564 T > G	Leu184Pro
		94,617 G > A	Asp202Asn
BHRF1	Early lytic phase (E)	42,157 A > T	Thr24Ser
		42,202 C > T	Arg39Cys
		42,322 G > T	Val79Leu
		42,349 T > G	Leu88Val
BaRF1	Early lytic phase (E)	66,966 G > A	Ala119Thr
BMRF1	Early lytic phase (E)	68,304 + 5 GT > TC	Val232Ser
		68,305 T > C	Val232Ala
		68,448 C > A	Leu280Met
		68,529 G > A	Ala307Thr
BMRF2	Early lytic phase (E)	69,430 G > A	Asp201Asn
		69,484 C > A	Leu219Met
		69,778 G > T	Ala317Ser
BSLF1	Early lytic phase (E)	72,169 G > C	Gln809Glu
		72,569 G > T	Asn675Lys
		72,865 C > T	Val577Ile
		73,063 C > G	Ala511Pro
		73,212 G > A	Thr461Met
		73,395 C > T	Arg400Gln
		73,726 T > C	Met290Val
		73,861 C > T	Ala245Thr
		74,190 G > A	Ala135Val
		74,219 C > A	Gln125His
		74,287 C > G	Asp103His
		74,304 C > T	Arg97His
		74,364 T > C	His77Arg
		74,587 C > T	Ala3Thr
BSRF1	Early lytic phase (E)	75,167 C > G	Pro178Ala
		75,288 C > T	Thr218Met
BBLF4	Early lytic phase (E)	99,562 G > A	Arg804Cys
		99,787 A > T	Ser729Thr
		99,948 T > C	Lys675Arg
		99,949 T > C	Lys675Glu
		100,036 G > T	His646Asn
		100,344 C > T	Arg543His
		100,354 G > T	Leu540Met
		100,725 G > T	Thr416Asn
		100,852 C > T	Ala374Thr
		100,894 T > C	Thr360Ala
		100,957 C > T	Ala339Thr
		101,008 G > T	Leu322Met
		101,183 C > A	Lys263Asn
		101,545 G > T	Leu143Ile
		101,692 T > A	Thr94Ser
		101,850 T > A	Gln41Leu
		101,862 C > T	Arg37Lys
		101,893 G > A	Arg27Cys
BBLF2/BBLF3	Early lytic phase (E)	104,536 G > A	Thr696Met
		104,550 A > C	Ile691Met
		104,555 T > C	Thr690Ala
		104,705 A > G	Ser640Pro
		104,824 T > C	His600Arg
		104,980 C > G	Gly548Ala
		105,023 C > T	Val534Met
		105,059 G > A	Arg522Trp
		105,244 C > T	Glu503Lys
		105,265 G > T	Pro496Thr
		106,044 C > T	Arg236His
		106,057 C > T	Ala232Thr
		106,060 C > T	Asp231Asn
		106,273 T > C	Met160Val
		106,293 A > C	Val153Gly
		106,375 T > G	Ile126Leu
BBLF1	Early lytic phase (E)	108,562 G > A	Ala42Val
BGLF5	Early lytic phase (E)	108,924 G > C	Pro377Arg
		109,776 T > G	Glu93Ala
		109,819 T > G	Lys79Gln
		109,917 A > T	Phe46Tyr
BGLF4	Early lytic phase (E)	110,123 G > A	Pro402Ser
		110,453 T > C	Thr292Ala
		110,934 C > A	Gln131His
BGLF3.5	Early lytic phase (E)	111,506 T > C	His54Arg
BGLF3	Early lytic phase (E)	111,718 C > T	Val312Ile
		112,041 T > C	Asn204Ser
		112,202 T > A	Gln150His
		112,396 G > A	Pro86Ser
BGRF1/BDRF1	Early lytic phase (E)	112,699 C > T	Ala17Val
		112,872 G > A	Val75Met
		112,882 C > T	Ala78Val
		112,981 A > T	Gln111Leu
		117,020 C > A	Gln344Lys
		117,051 C > T	Ser354Phe
		117,588 T > C	Val533Ala
		117,837 G > T	Ser616Ile
BDLF3.5	Early lytic phase (E)	116,795 C > G	Glu51Gln
		116,822 C > T	Val42Met
		116,840 T > C	Thr36Ala
		116,885 C > T	Glu21Lys
		116,902 C > A	Gly15Val
		116,935 G > A	Pro4Leu
BcRF1	Early lytic phase (E)	125,273 A > G	Thr33Ala
		125,385 G > A	Arg70Gln
		125,389 G > A	Met71Ile
		125,574 A > G	Asn133Ser
		125,688 G > A	Arg171Gln
		125,718 A > C	Asn181Thr
		125,719 T > G	Asn181Lys
		125,868 C > T	Thr231Ile
		125,894 C > G	Leu240Val
		126,375 C > T	Ser400Phe
		126,819 G > A	Arg548Gln
		126,882 A > G	Gln569Arg
		127,409 T > C	Cys745Arg
BTRF1	Early lytic phase (E)	127,821 G > A	Ala136Thr
		127,992 G > A	Ala193Thr
		128,037 A > G	Thr208Ala
		128,067 G > A	Gly218Arg
		128,160 C > G	Pro249Ala
		128,269 C > A	Pro285Gln
		128,473 T > G	Ile353Ser
BALF5	Early lytic phase (E)	153,360 C > T	Asp977Asn
		153,543 T > C	Thr916Ala
		153,706 G > T	His861Gln
		153,961 A > C	Asp776Glu
		154,053 C > T	Glu746Lys
		154,847 G > A	Ala481Val
		155,085 A > T	Ser402Thr
		155,288 C > T	Arg334His
		155,352 C > T	Asp313Asn
		155,609 C > T	Arg227Gln
		155,829 T > G	Ser154Arg
BALF2	Early lytic phase (E)	161,018 A > G	Ser1099Pro
		161,036 T > C	Ser1093Gly
		161,114 C > T	Ala1067Thr
		161,117 C > T	Ala1066Thr
		161,119 T > C	Glu1065Gly
		161,122 G > A	Ala1064Val
		162,215 C > A	Val700Leu
		162,476 T > C	Ile613Val
		163,066 A > G	Leu416Pro
		163,364 C > T	Val317Met
		163,438 G > A	Ser292Leu
		163,772 A > C	Ser181Ala
BALF1	Early lytic phase (E)	164,674 C > T	Arg91Gln
		164,678 C > T	Val90Ile
		164,903 G > A	Pro15Ser
BARF1	Early lytic phase (E)	165,131 T > C	Val29Ala
BNLF2b	Early lytic phase (E)	166,625 A > C	Phe71Cys
		166,632 G > T	Gln69Lys
		166,781 C > G	Arg19Thr
		166,797 G > C	Leu14Val
		166,802 G > T	Ala12Asp
		166,829 G > T	Pro3Gln
BNLF2a	Early lytic phase (E)	166,943 C > T	Ser29Asn
BNRF1	Late lytic phase (L)	1769 G > A	Val12Ile
		1841 G > C	Glu36Gln
		1917 T > A	Leu61Gln
		1928 G > A	Ala65Thr
		2135 C > G	Gln134Glu
		2333 G > C	Gly200Arg
		2544 T > G	Leu270Trp
		2573 A > G	Thr280Ala
		3101 G > A	Gly456Arg
		3188 G > T	Ala485Ser
		3191 A > G	Arg486Gly
		3224 A > G	Ser497Gly
		3380 G > A	Gly549Ser
		3452 T > A	Phe573Ile|
		3816 C > A	Pro694His
		3942 G > T	Cys736Phe
		4014 A > C	Asn760Thr
		4019 G > T	Ala762Ser
		4125 A > G	Asn797Ser
		4567 G > A	Met944Ile
		4947 G > A	Arg1071His
		4951 G > C	Glu1072Asp
		5064 T > C	Phe1110Ser
		5081 G > T	Ala1116Ser
		5399 G > A	Val1222Ile
		5600 G > A	Ala1289Thr
BCRF1	Late lytic phase (L)	9690 G > A	Val6Met
		9741 G > A	Gly23Ser
		9875 T > G	Phe67Leu
BFLF2	Late lytic phase (L)	43,965 A > G	Ile228Thr
		44,005 T > G	Ile215Leu
		44,461 T > G	Ser63Arg
		44,590 C > T	Ala20Thr
		44,590 C > A	Ala20Ser
		44,627 A > C	Asp7Glu
BFLF1	Late lytic phase (L)	45,489 A > T	Leu250Gln
		45,502 C > T	Val246Ile
		46,106 G > C	Asp44Glu
		46,132 C > T	Ala36Thr
		46,134 A > G	Ile35Thr
		46,192 C > T	Gly16Arg
BFRF1A	Late lytic phase (L)	46,368 C > T	Leu45Phe
		46,410 C > A	Leu59Ile
		46,611 C > T	Pro126Ser
BFRF1	Late lytic phase (L)	47,035 G > A	Asp145Asn
		47,237 C > A	Pro212His
		47,239 G > A	Gly213Ser
		47,273 A > T	His224Leu
		47,539 G > A	Ala313Thr
		47,596 T > G	Phe332Val
BFRF2	Late lytic phase (L)	47,539 G > A	Arg7His
		47,568 T > G	Cys17Gly
		47,590 C > T	Pro24Leu
		47,596 T > G	Val26Gly
		47,599 C > T	Ser27Phe
		47,620 A > C	Gln34Pro
		47,638 G > A	Gly40Glu
		47,905 T > C	Val129Ala
		47,907 G > T	Val130Phe
		48,025 A > C	Asp169Ala
		48,609 C > T	Arg364Trp
		48,862 G > T	Cys448Phe
		48,904 G > A	Arg462Gln
		48,954 C > T	Arg479Cys
		49,126 C > T	Ala536Val
		49,140 C > T	His541Tyr
		49,242 C > T	His575Tyr
BFRF3	Late lytic phase (L)	49,713 C > A	His165Gln
		49,724 C > T	Pro169Leu
BPLF1	Late lytic phase (L)	49,987 C > T	Gly3085Ser
		50,134 G > C	Gln3036Glu
		50,241 C > T	Arg3000Gln
		50,254 T > C	Thr2996Ala
		50,291 C > A	Glu2983Asp
		50,446 C > T	Ala2932Thr
		50,907 C > T	Gly2778Asp
		50,947 G > A	Pro2765Ser
		50,952 C > G	Ser2763Thr
		51,080 A > C	Ser2720Arg
		51,222 C > T	Arg2673Gln
		51,228 C > A	Gly2671Val
		51,270 A > G	Val2657Ala
		51,276 C > A	Arg2655Leu
		51,322 C > A	Ala2640Ser
		51,394 T > C	Lys2616Glu
		51,436 G > A	Arg2602Trp
		51,570 G > T	Pro2557Gln
		51,586 C > T	Glu2552Lys
		51,589 G > T	His2551Asn
		51,664 C > T	Asp2526Asn
		51,888 A > T	Leu2451Gln
		52,071 C > T	Arg2390Lys
		52,474 G > A	Arg2256Cys
		52,495 C > T	Glu2249Lys
		52,504 T > C	Asn2246Asp
		52,705 C > T	Ala2179Thr
		52,709 C > T	Met2177Ile
		52,740 C > A	Arg2167Leu
		52,861 C > A	Ala2127Ser
		53,415 T > A	Tyr1942Phe
		53,430 G > A	Ser1937Leu
		53,605 C > A	Val1879Phe
		53,770 C > G	Gly1824Arg
		53,772 G > A	Ala1823Val
		53,845 C > T	Ala1799Thr
		53,986 G > A	Arg1752Cys
		54,219 G > A	Ser1674Leu
		54,339 A > G	Ile1634Thr
		54,364 C > A	Ala1626Ser
		54,504 T > C	Asp1579Gly
		54,505 C > T	Asp1579Asn
		54,597 G > T	Pro1548His
		54,635 A > C	Asp1535Glu
		54,639 G > A	Thr1534Met
		54,705 C > T	Arg1512Gln
		54,821 G > T	Asp1473Glu
		54,957 T > C	Gln1428Arg
		55,230 G > A	Thr1337Met
		55,297 T > C	Ile1315Val
		55,633 T > C	Ile1203Val
		55,738 C > T	Asp1168Asn
		56,432 T > A	Glu936Asp
		56,485 T > G	Asn919His
		56,508 T > C	Glu911Gly
		56,511 C > T	Gly910Glu
		56,514 G > C	Ala909Gly
		56,569 G > T	Leu891Met
		56,586 T > C	Asp885Gly
		56,644 C > T	Val866Ile
		56,655 G > A	Thr862Met
		56,701 T > C	Lys847Glu
		56,854 A > C	Ser796Ala
		57,176 C > G	Glu688Asp
		57,198 G > A	Ala681Val
		57,271 C > T	Val657Ile
		57,280 A > C	Ser654Ala
		58,386 C > T	Gly285Asp
		59,164 C > T	Val26Met
		59,188 C > T	Val18Met|
		59,206 T > G	Thr12Pro
BOLF1	Late lytic phase (L)	59,246 C > T	Ala1236Thr
		59,704 G > A	Ala1083Val
		59,833 A > T	Val1040Asp
		59,849 G > A	Pro1035Ser
		59,888 G > A	Pro1022Ser
		59,966 T > G	Ser996Arg
		59,981 G > T	Leu991Met
		60,028 G > A	Pro975Leu
		60,049 A > G	Ile968Thr
		60,265 T > C	His896Arg
		60,454 G > T	Ser833Tyr
		60,527 T > C	Arg809Gly
		60,568 C > T	Arg795Lys
		60,861 C > A	Glu697Asp
		60,881 C > A	Ala691Ser
		60,888 T > G	Glu688Asp
		60,894 C > A	Glu686Asp
		61,514 C > T	Ala480Thr
		62,344 G > A	Ala203Val
		62,500 G > T	Ala151Glu
		62,501 C > T	Ala151Thr
		62,520 C > A	Glu144Asp
		62,924 T > C	Ser10Gly
BORF1	Late lytic phase (L)	63,055 A > G	Thr36Ala
		63,689 T > G	Val247Gly
		63,965 A > T	Asn339Ile
BORF2	Late lytic phase (L)	65,068 A > C	Asp317Ala
		65,376 G > T	Ala420Ser
		65,466 G > A	Val450Ile
		66,318 G > A	Asp734Asn
		66,406 C > T	Pro763Leu
		66,435 G > A	Ala773Thr
BLLF3	Late lytic phase (L)	75,430 T > C	Ser253Gly
		75,448 T > C	Thr247Ala
		75,484 G > T	Leu235Ile
		75,622 C > T	Gly189Ser
BLRF1	Late lytic phase (L)	76,499 A > G	Ile81Val
BLRF2	Late lytic phase (L)	77,037 C > T	Pro134Leu
		77,045 T > C	Ser137Pro
BLLF1	Late lytic phase (L)	77,206 C > T	Arg887His
		77,387 T > C	Lys827Glu
		77,393 A > G	Ser825Pro
		77,432 G > T	Pro812Thr
		77,452 C > T	Gly805Asp
		77,606 G > T	Gln754Lys
		77,641 G > A	Pro742Leu
		77,734 C > T	Ser711Asn
		77,737 A > G	Ile710Thr
		78,544 T > G	Asp441Ala
		78,547 G > T	Ala440Asp
		78,565 A > G	Leu434Ser
		79,111 G > A	Ala252Val
		79,221 A > T	Asp215Glu
		79,265 C > G	Glu201Gln
BLLF2	Late lytic phase (L)	77,325 T > A	Gln134Leu
		77,418 C > T	Arg103Gln
		77,432 G > T	Asp98Glu
		77,452 C > T	Val92Met
		77,641 G > A	Arg29Trp
		77,703 T > C	His8Arg
BKRF2	Late lytic phase (L)	97,676 G > A	Ala3Thr
		97,698 T > C	Ile10Thr
		97,973 G > A	Gly102Ser
		98,006 A > T	Thr113Ser
BKRF3	Late lytic phase (L)	98,252 C > T	Thr63Ile
		98,377 G > A	Val105Ile
		98,423 C > T	Ala120Val
BKRF4	Late lytic phase (L)	98,904 C > T	Ser20Leu
		98,985 T > C	Val47Ala
		99,022 -GAG	Glu61del
		99,107 G > A	Asp88Asn
		99,110 T > G	Ser89Ala
		99,282 C > T	Pro146Leu
		99,330 A > C	Gln162Pro
		99,351 G > T	Gly169Val
		99,356 C > A	His171Asn
BBRF1	Late lytic phase (L)	102,198 G > A	Asp95Asn
		102,385 C > A	Pro157His
		102,481 C > T	Thr189Met
		102,502 A > G	Lys196Arg
		102,512 A > G	Ile199Met
		102,600 T > G	Cys229Gly
		103,587 A > G	Ser558Gly
		103,645 C > T	Ala577Val
		103,695 A > G	Thr594Ala
BBRF2	Late lytic phase (L)	103,825 A > G	Ile56Val
		103,885 G > A	Val76Ile
BBRF3	Late lytic phase (L)	106,891 G > T	Val15Phe
		106,898 C > T	Thr17Met
		107,458 G > A	Val204Ile
		107,593 A > G	Ile249Val
		107,644 C > A	Leu266Met
		107,784 C > G	Ile312Met
		108,013 G > A	Val389Ile
BGLF2	Late lytic phase (L)	113,774 G > T	Pro271His
		113,915 C > T	Arg224His
		114,449 A > G	Val46Ala
BGLF1	Late lytic phase (L)	114,763 G > A	Pro442Ser
		114,864 -TAG	Thr407del
		115,296 A > G	Leu264Pro
		115,324 C > T	Gly255Ser
		115,411 A > G	Ser226Pro
		115,488 G > A	Ala200Val
		115,575 A > G	Val171Ala
		115,640 T > C	Ile149Met
		115,712 C > G	Arg125Ser
		115,832 A > C	Asp85Glu
BDLF4	Late lytic phase (L)	116,237 G > C	Ala166Gly
		116,311 C > A	Glu141Asp
		116,331 C > T	Glu135Lys
		116,394 T > G	Lys114Gln
		116,502 A > C	Tyr78Asp
		116,568 C > T	Asp56Asn
		116,670 G > A	Arg22Cys
BDLF3	Late lytic phase (L)	118,154 C > T	Ala209Thr
		118,745 T > G	Met12Leu
BDLF2	Late lytic phase (L)	119,032 C > G	Cys357Ser
		119,170 C > T	Arg311Gln
		119,272 G > A	Ala277Val
		119,296 T > A	His269Leu
		119,789 T > C	Thr105Ala
		119,867 G > C	Pro79Ala
		119,882 G > A	Pro74Ser
BDLF1	Late lytic phase (L)	120,867 G > T	Leu51Ile
BcLF1	Late lytic phase (L)	121,730 C > T	Arg1150His
		122,141 A > G	Val1013Ala
		122,257 A > T	Asn974Lys
		122,436 C > T	Ala915Thr
		122,444 G > T	Pro912Gln
		122,463 C > T	Val906Ile
		122,490 C > T	Ala897Thr
		123,228 A > C	Ser651Ala
		123,636 C > T	Val515Met
		124,122 G > T	Pro353Thr
		124,560 A > C	Phe207Val
		124,884 C > T	Val99Ile
BXLF2	Late lytic phase (L)	129,234 G > A	Ala505Val
		129,249 G > A	Thr500Met
		129,288 G > T	Thr487Lys
		130,159 C > T	Val197Ile
		130,270 G > T	Leu160Met
		130,647 G > A	Ser34Leu
BXLF1	Late lytic phase (L)	131,183 T > G	Ser464Arg
		131,282 T > C	Thr431Ala
		131,614 C > A	Arg320Leu
		131,776 A > G	Met266Thr
		131,992 C > T	Arg194His
		132,175 C > A	Arg133Leu
		132,511 T > A	Asp21Val
BXRF1	Late lytic phase (L)	132,830 A > C	Tyr87Ser
BVRF1	Late lytic phase (L)	134,120 G > T	Ala332Ser
		134,139 G > T	Gly338Val
		134,147 G > T	Gly341Cys
		134,372 A > G	Met416Val
		134,402 C > T	Pro426Ser
		134,475 C > T	Ala450Val
		134,672 C > G	Gln516Glu
BVLF1	Late lytic phase (L)	134,887 C > G	Glu247Asp
		135,002 G > A	Ala209Val
BVRF2	Late lytic phase (L)	135,831 C > T	Ala65Val
		135,867 A > T	Asp77Val
		135,950 G > T	Val105Leu
		136,646 G > T	Ala337Ser
		137,081 T > C	Ser482Pro
		137,255 G > A	Ala540Thr
		137,271 C > T	Pro545Leu
		137,316 A > C	His560Pro
		137,327 A > C	Lys564Gln
BdRF1	Late lytic phase (L)	136,646 G > T	Ala337Ser
		137,081 T > C	Ser482Pro
		137,255 G > A	Ala540Thr
		137,271 C > T	Pro545Leu
		137,316 A > C	His560Pro
		137,327 A > C	Lys564Gln
BILF2	Late lytic phase (L)	137,734 G > A	Ser168Phe
		137,809 + 10 GC > CA	Ala143Cys
		137,811 A > T	His142Gln
		137,882 G > T	Leu119Met
		138,014 T > G	Thr75Pro
		138,035 C > G	Val68Leu
		138,154 A > G	Val28Ala
LF3	Late lytic phase (L)	140,573 G > C	Phe924Leu
		140,601 T > G	Lys915Thr
LF2	Late lytic phase (L)	149,042 G > C	Thr428Ser
		149,319 C > T	Val336Ile
		149,355 C > T	Val324Ile
		150,212 A > G	Met38Thr
		150,218 G > A	Ser36Leu
LF1	Late lytic phase (L)	150,358 G > A	Pro446Leu
		150,725 G > T	Leu324Met
		150,754 G > A	Ser314Phe
		150,778 G > A	Pro306Leu
		150,823 C > T	Arg291Lys
		150,872 A > T	Ser275Thr
		150,967 C > T	Arg243Gln
		150,974 C > T	Ala241Thr
		150,994 C > T	Gly234Glu
		151,078 A > G	Val206Ala
		151,140 G > T	Asn185Lys
		151,147 G > T	Ser183Tyr
		151,256 G > T	Gln147Lys
		151,337 C > A	Ala120Ser
		151,671 C > A	Gln8His
		151,678 T > C	Asp6Gly
BILF1	Late lytic phase (L)	152,578 T > C	Asn22Asp
		152,625 G > A	Ala6Val
BALF4	Late lytic phase (L)	156,491 T > C	Thr792Ala
		156,637 G > A	Ala743Val
		158,143 T > G	Lys241Thr
		158,144 T > C	Lys241Glu
		158,408 T > C	Thr153Ala
		158,481 G > C	Asp128Glu
		158,747 T > C	Thr40Ala
BALF3	Late lytic phase (L)	159,696 T > G	Ser405Arg
		159,712 C > G	Glu399Asp
		159,960 C > G	Gly317Arg
		160,331 T > A	Glu193Val
		160,842 T > C	Thr23Ala
BARF0	Latent phase and lytic phase	159,133 G > A	Ala5Thr
		159,208 T > C	Cys30Arg
		159,220 G > A	Gly34Arg
		159,301 G > A	Gly61Arg
		159,604 G > A	Ala162Thr
		159,712 C > G	Leu198Val
		159,760 C > G	Gln214Glu
		159,769 C > T	Pro217Ser
		159,889 C > T	Arg257Cys
		159,907 G > A	Gly263Ser
		159,910 G > A	Val264Ile
		160,015 G > T	Ala299Ser
		160,057 G > A	Ala313Thr
		160,087 C > T	Leu323Phe
		160,243 C > A	Arg375Ser
		160,249 G > C	Ala377Pro
		160,249 G > T	Ala377Ser
		160,331 T > A	Glu193Val

#### Comparison of variant sites across EBV-associated disease subtypes

**Fig. 2:**
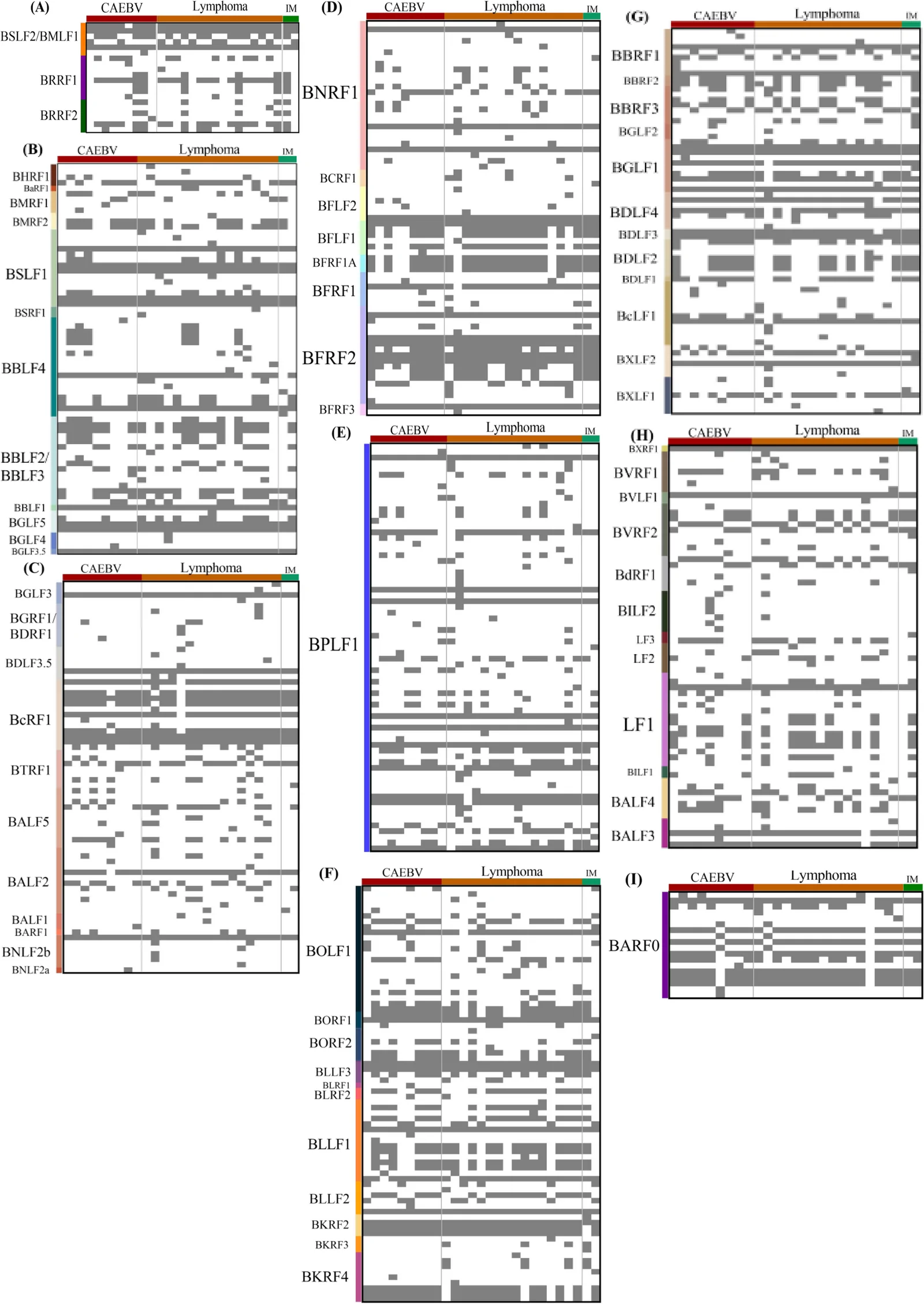
Variant sites of EBV DNA based on EBV-associated disease subtypes. (**A**) encoding proteins expressed in immediately early phase; (**B**) encoding proteins expressed in early phase; (**C**) encoding proteins expressed in late phase; (**D**) encoding proteins expressed in both early and late phase

Patients were categorized into three groups based on their EBV-associated disease subtype: IM, CAEBV or EBV^+^NK/T lymphoma groups. Heatmaps were generated to visualize variant profiles across groups (Fig. 2), and comparative analysis of individual variant sites revealed no statistically significant differences. These results indicate that single-amino-acid changes in EBV proteins are not a major determinant of disease subtype classification.

**Fig. 3:**
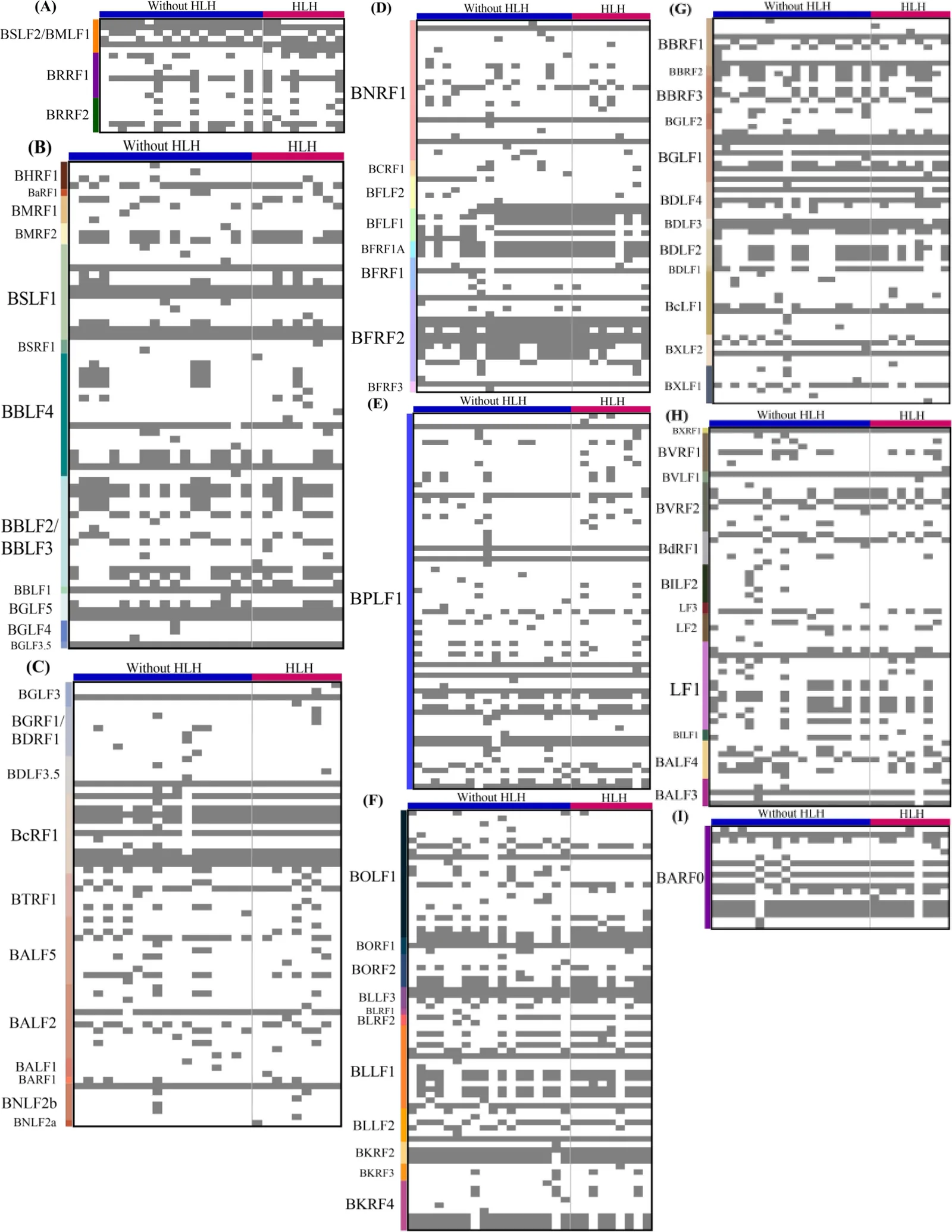
Variant sites of EBV DNA based on comorbidity with HLH. (**A**) encoding proteins expressed in immediately early phase; (**B**) encoding proteins expressed in early phase; (**C**) encoding proteins expressed in late phase; (**D**) encoding proteins expressed in both early and late phase

The incidence of concurrent HLH varied among the disease subtypes. No HLH cases were observed in the IM group, while the incidence rate was 2/7 in the CAEBV group. Notably, lymphoma patients exhibited a significantly higher rate of 7/16 (Table 1). To examine whether EBV sequence variation is associated with HLH development, variant profiles were compared between patients with and without HLH (Fig. 3). No statistically significant variant sites were identified, indicating that single-amino-acid substitutions in EBV proteins are not linked to HLH occurrence in this cohort.

#### Comparison of variant sites with clinical outcomes

Disease severity and clinical outcomes vary considerably among the three EBV-associated diseases included in this study: IM, CAEBV and EBV-positive NK/T-cell lymphoma. While IM typically follows a mild course with most patients recovering spontaneously, CAEBV and EBV-positive NK/T-cell lymphoma frequently involve severe disease progression. These conditions are often characterized by widespread infiltration of proliferating lymphocytes or tumor cells into multiple organs, leading to life-threatening organ dysfunction. Based on hospital records and follow-up data, all IM patients in our cohort achieved remission or were completely cured. In contrast, the remission rate was 0% in the CAEBV group and 18.8% in the lymphoma group, respectively. Mortality was notably high, reaching 22.2% in CAEBV patients and 43.8% in lymphoma patients.

**Fig. 4:**
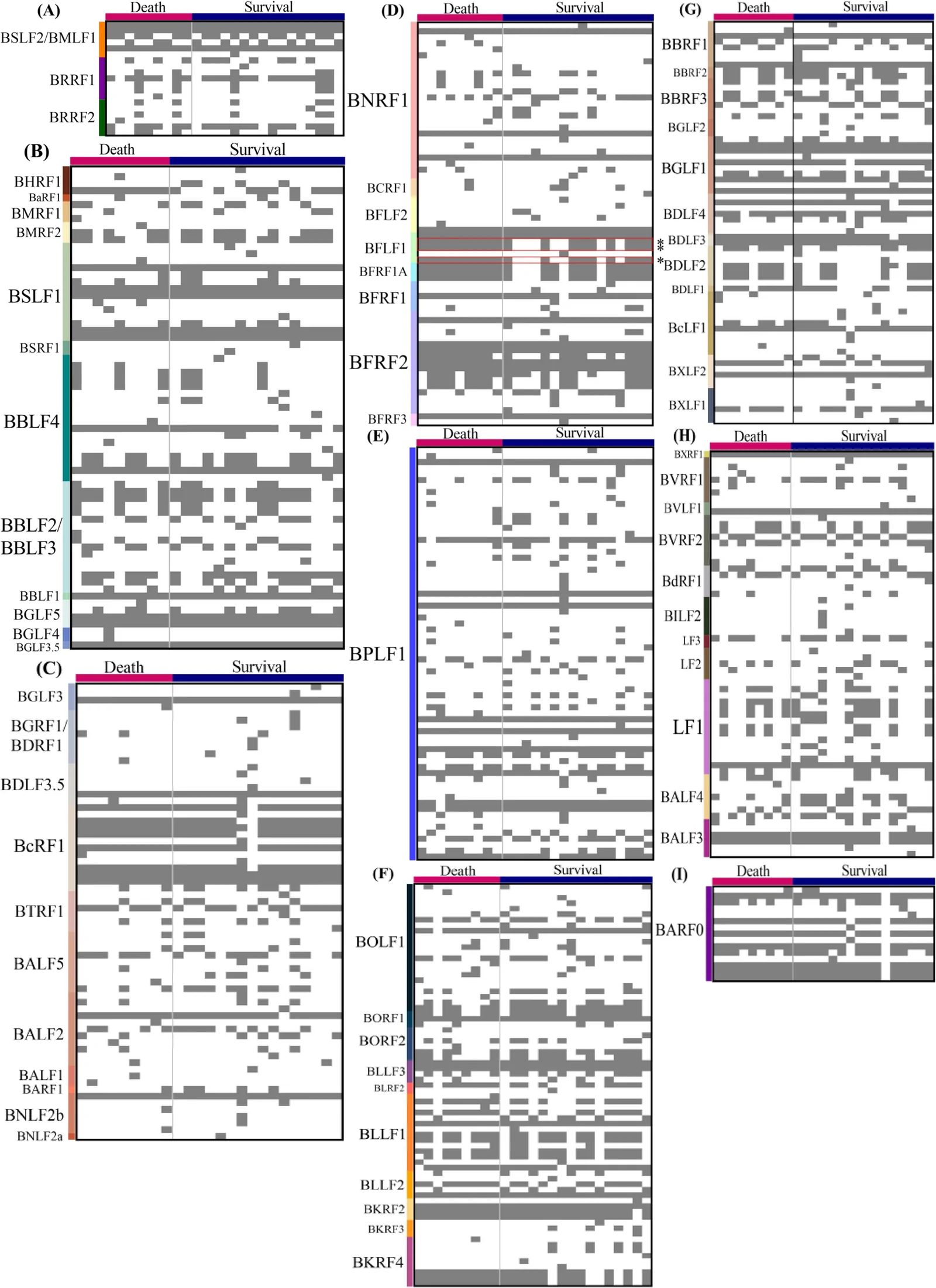
Variant sites of EBV DNA based on clinical outcomes. (**A**) encoding proteins expressed in immediately early phase; (**B**) encoding proteins expressed in early phase; (**C**) encoding proteins expressed in late phase; (**D**) encoding proteins expressed in both early and late phase

**Fig. 5:**
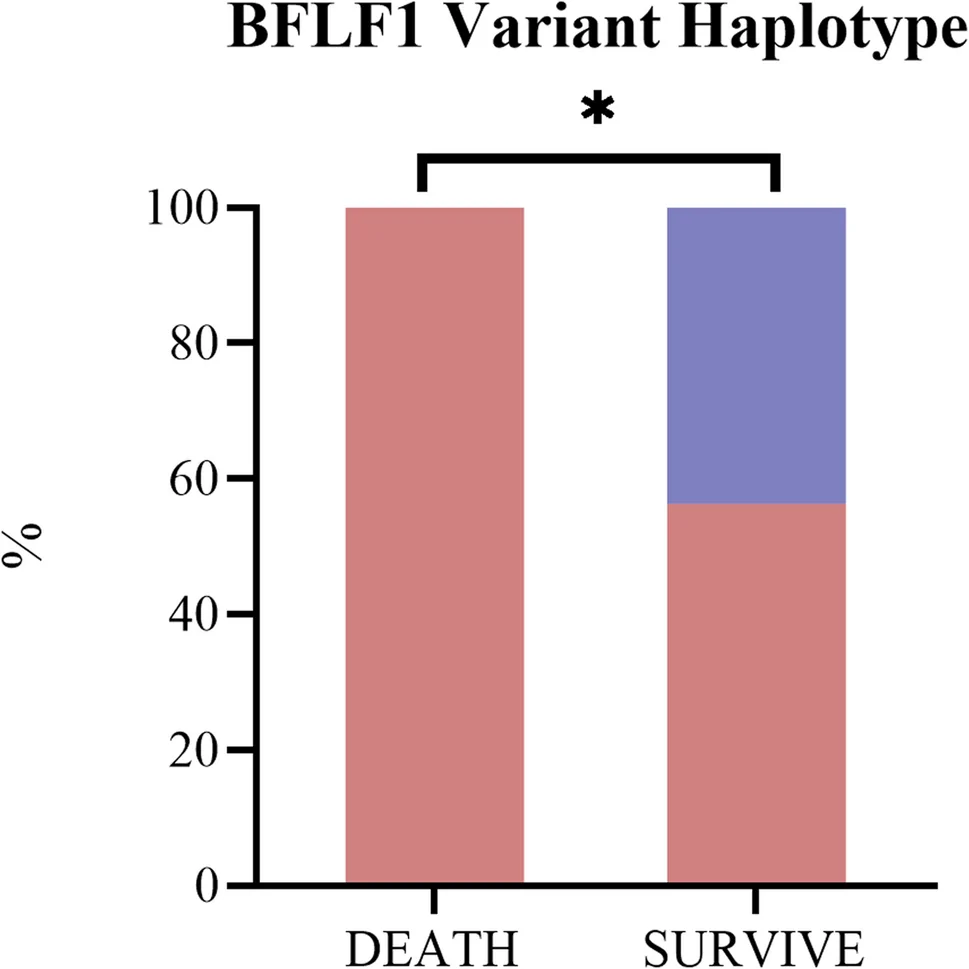
The frequency of mutant amino acid in BFLF1 between dead and live patients

To evaluate whether EBV sequence variation influences clinical outcomes, we compared genomic polymorphisms across the study population (Fig. 4). After excluding two patients lost to follow-up, three specific variants in the BFLF1 gene were found to be significantly associated with mortality: nucleotide changes at positions 104, 132, and 736, resulting in amino acid substitutions at protein positions 35 (from Ile to Thr), 44 (from Asp to Glu), and 246 (from Val to Ile), respectively. These variants were significantly more frequent in deceased patients than in survivors (*P* = 0.0267) (Fig. 5). The corresponding odds ratio (OR) was as high as 15.00 [95% CI = 0.7436, 301.5].

To exclude statistical bias caused by disease types, we tabulated the BFLF1 mutation frequencies across the distinct disease tracks (excluding 2 patients lost to follow-up). In the IM and CAEBV group, the BFLF1 variant rate was 66.7% (6 out of 9 patients) and in the Extranodal NK/T-cell Lymphoma group 75.0% (12 out of 16 patients). Statistically, there is no significant difference in the prevalence of these BFLF1 variants between the benign and malignant groups.

### Structural anylysis of mutant BFLF1

**Fig. 6:**
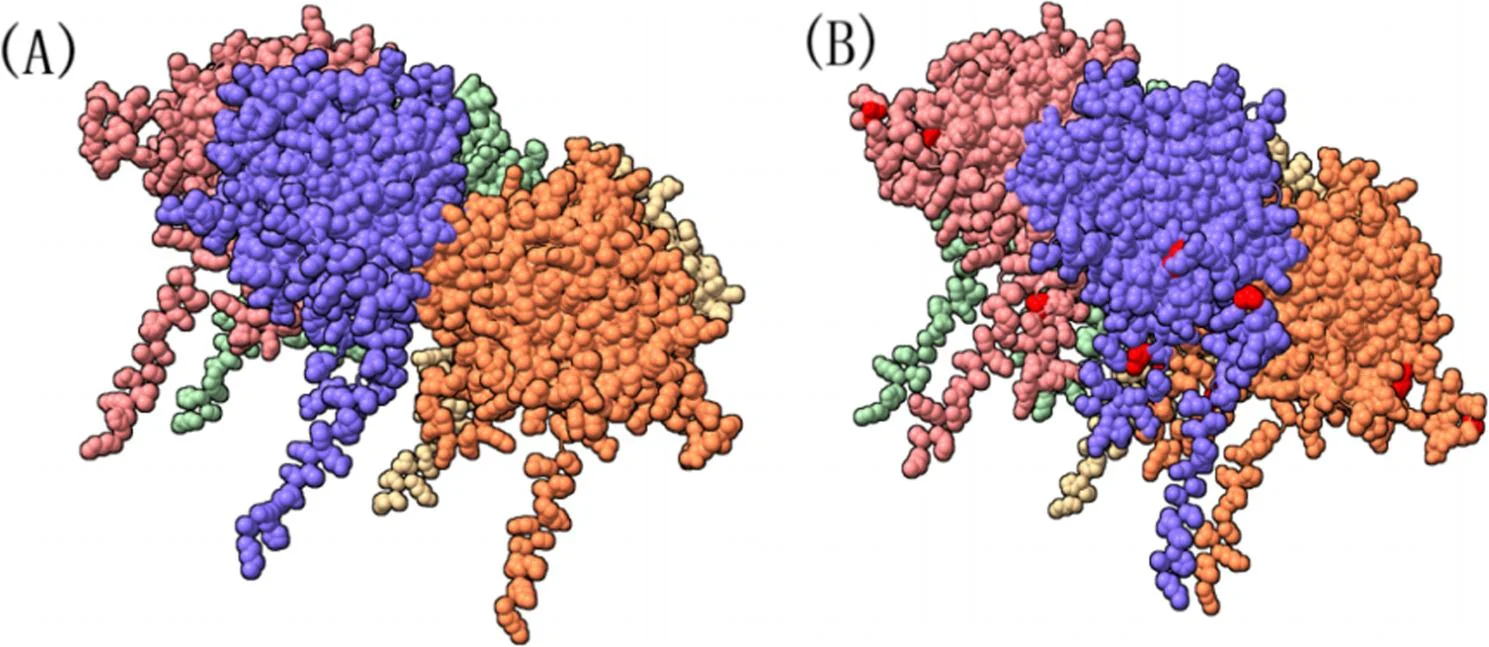
Structure of BFLF1 predicted by AlphaFold3. (**A**) wildtype BFLF1 subunit; (**B**) mutant BFLF1 subunit. *Red spots highlight the mutant amino acids

Given that these three missense variant sites within the BFLF1 gene exhibited complete linkage disequilibrium—either co-occurring concurrently as a single haplotypic block or remaining entirely absent across all analyzed genomes—we hypothesized that they represent a highly conserved, distinct synchronized variant pattern associated with clinical outcomes. To evaluate the potential structural impact, we introduced all three single amino acid substitutions simultaneously into a mutant protein model. According to Uniprot, the native BFLF1 protein consists of two subunits, each assembled from five identical polypeptide chains. Due to the current limitation of AlphaFold3, we ultimately generated one of these two subunits. The structure was visualized using ChimeraX (Fig. 6), with the three substituted amino acid positions marked in red. Comparison between the wild type and mutant models revealed no apparent structural alterations, suggesting that these amino acid changes have a negligible effect on the overall conformation of the BFLF1 protein. At the sub-residue level, the three linked substitutions introduce distinct localized physicochemical shifts. The 44 (from Asp to Glu) and 246 (from Val to Ile) substitutions represent highly conservative changes, strictly maintaining the hydrophobic aliphatic and negatively charged properties of their respective wild-type residues. In contrast, the 35 (from Ile to Thr) substitution introduces a localized chemical transition, substituting a highly hydrophobic amino acid (Isoleucine) with a hydrophilic residue (Threonine) at position 35.

## Discussion

EBV-associated diseases encompass a broad spectrum ranging from benign proliferative disorders to highly malignant tumors. Their heterogeneity poses significant challenges for clinical diagnosis, treatment selection, and prognosis assessment. Our research focuses on analyzing how genetic polymorphisms in EBV sequence influence the complex pathogenesis and clinical outcomes of EBV-associated diseases. Tiny disparity was observed among patients with different types of EBV-associated diseases. Only 3 sites on BFLF1 encoding gene were found to be more frequent in EBV from patients with poor outcomes.

The development of EBV-associated diseases involves the transformation and proliferation of EBV-infected lymphocytes, coupled with an imbalanced host immune surveillance against viral-infected cells. Various proteins encoded by EBV exhibit immunosuppressive effects. For instance, upon the establishment of long-term latency, the persistent EBNA1 functions to inhibit Toll-like receptor (TLR) pathways; conversely during late lytic replication, the virion-associated tegument protein BPLF1 acts swiftly to suppress retinoic acid-inducible gene I (RIG-I) and interferon (IFN) signaling pathways. These mechanisms enable EBV to evade immune defense and surveillance, achieving prolonged latency and short-term lytic replication in infected cells [10]. Additionally, acquired immune deficiencies caused by human immunodeficiency virus (HIV) infection or immunosuppressive agents, along with inborn errors of immunity (IEIs), all increase susceptibility to EBV-associated diseases [11].

EBV can exist in host cells through multiple latent modes, expressing distinct latent genes such as EBNA1 (new BKRF1), EBNA2 (new BYRF1), LMP1 (new BNLF1), and LMP2A/B. These genes play crucial roles in maintaining viral latency, promoting cell proliferation, inhibiting apoptosis and evading immune clearance. During the lytic activation and replication phase, EBV undergoes a highly regulated cascade of gene expression dividing into immediate-early (IE), early (E), and late (L) phases. This replication cascade initiates with the expression of IE transcription factors (such as BZLF1), which act as master molecular switches to transactivate E genes involved in the viral replication machinery, including the DNA polymerase (BALF5) and the viral protein kinase (BGLF4). This is followed chronologically by the expression of L genes encoding viral structural proteins required for virion assembly and maturation (such as BFLF1). This stringently coordinated kinetic cascade ensures successful lytic replication and propagation within host cells. The differences in EBV gene expression patterns, variations in host immune status, and diversity in affected lymphocyte types collectively shape the remarkable heterogeneity of EBV-associated diseases. By comparing EBV genomes derived from 27 patients with the reference sequence B95.8/Raji, we identified 2159 single nucleotide variants (SNVs), 12 insertion, and 11 deletion. Most (1636 SNVs, 11 insertions, and 9 deletions) were located in regulatory regions (e.g. upstream/downstream of coding frames or intergenic regions), with no direct amino acid alteration in protein. These findings indicate that EBV sequences in EBV-associated diseases patients remain relatively conserved, and genomic polymorphisms do not lead to protein structural diversity.

Despite the limited number of variants detected in the reading frame, the 523 missense variants, 1 insertion, and 2 deletion involve single amino acid alterations in 74 EBV structural proteins with multiple functions. For example, BNRF1, BPLF1 and BKRF4 are tegument proteins located between the capsid and envelope, playing crucial roles in nucleic acid transportation, virus assembly and immune evasion [12]. Notably, the rest 73 proteins except BARF0 with detected amino acid changes were expressed during the lytic phase, suggesting a close relationship between genomic changes in lytic phase proteins and EBV-associated diseases pathogenesis. This striking enrichment suggests a compelling coordination between genomic variations in lytic-phase proteins and the pathogenesis of specific EBV-associated diseases. Historically, researchers have frequently focused on classical latency genes, such as BNLF1 and BKRF1, as primary mutational hotspots in EBV-related malignancies. For instance, Borozan et al. [13], on the other hand, detected 13 novel sequences in the EBV genomes of patients with EBV-associated gastric adenocarcinoma. Their comparison with B95.8/Raji revealed that variant sites were predominantly concentrated in latency genes, highlighting the role of these genes in carcinogenesis. Khine et al. [14] also identified recurrent EBNA3B deletions as a tumor suppressor based on their global population cohort. However, in our cohort focusing on hematopoietic malignancies and lymphoproliferative disorders, variants within these classical latency loci were remarkably absent. This pathogenic profile highlights a potential “lytic-to-latency” driving mechanism unique to lymphocyte-infiltrating diseases. Muruta et al. [15] noted that genome deletion causes overexpression of lytic phase genes, while EBV’s failure to complete its lysis due to missing key genes leads to persistent infection in host cells, ultimately promoting the development of EBV-associated diseases such as CAEBV. Polymorphisms in lytic replication proteins may alter viral replication kinetics or induce low-level spontaneous reactivation, which continuously expands the reservoir of latently infected NK/T or B cells and sustains host microenvironmental inflammation. Variants in lytic genes can also, though slightly, influence other EBV-related diseases. Xu et al. [16] identified two BALF2 variant sites with higher risk of nasopharyngeal carcinoma through genomic sequencing of patients with nasopharyngeal carcinoma, other EBV-associated diseases and healthy individuals, providing a valuable tool for screening nasopharyngeal carcinoma. Due to insufficient sample size and limited disease spectrum, we failed to identify statistically significant sites from the discovered variants. Nevertheless, this finding suggests that EBV in EBV-associated disease patients, which promotes pathological proliferation of lymphocytes rather than epithelial cells, possesses distinct genetic sequences compared to other EBV-associated epithelial malignancies.

Although not finding any difference between disease types and the risk of HLH development, our comparison of patients with different clinical outcomes revealed that variants at three sites in the BFLF1 gene were significantly more prevalent in death patients than in survival patients. This suggests that changes in the BFLF1 protein structure may be positively related to the mortality risk in EBV-associated diseases. The BFLF1 protein, also known as the DNA packaging protein UL32, plays a crucial role in the herpesviral life cycle by participating in the cleavage and packaging of the viral genome. Its main function is to encapsulate newly replicated viral DNA into the capsid, thereby forming mature viral particles [17]. Pavlova et al. [18] successfully constructed an EBV mutant devoid of BFLF1 which produced virus-like particles (VLPs) free of EBV DNA that elicited a strong CD4^+^ T cell response. It highlights the essential role of BFLF1 in the assembly of EBV virion structure. While the role of UL32 has been extensively studied in other herpesviruses, there is a remarkable lack of research focusing on the specific function of BFLF1 in EBV-associated diseases. Our finding, therefore, assists to fill this gap in the literature.

Given the global prevalence of EBV, we aimed to investigate regional differences in EBV strains and their impact on EBV-associated diseases through phylogenetic analysis. EBV-associated tumors including nasopharyngeal carcinoma, gastric cancer, and lymphoma exhibit significant regional variations. A 2020 epidemiological study revealed that between 1990 and 2017, East Asia accounted for 43% of EBV-related tumor cases and 40% of related deaths, because of the high prevalence of nasopharyngeal and gastric cancers in East Asia particularly China [19]. Palser et al. [20] analyzed EBV DNA sequences from 71 healthy individuals and EBV-associated tumor patients across multiple countries and regions, covering East Asia, Western Europe, Central Africa, North America and Oceania. They identified genetic variations concentrated in latency genes such as EBNA2 and LMP1, which may be crucial for distinguishing asymptomatic EBV infections from pathogenic cases. Our study enrolled patients from five eastern Chinese provinces (Shanghai, Jiangsu, Zhejiang, Anhui, Jiangxi) and western Xinjiang. Through phylogenetic analysis, we found that the results did not demonstrate significant regional differences due to insufficient geographical diversity of the included patients. Lin et al. [21] detected EBV sequences in the peripheral blood of 8 Chinese patients with extranodal NK/T cell lymphoma (nasal type) (NKTCL). The phylogenetic tree also confirmed that their EBV sequences were similar to those of other Asian NKTCL patients, further indicating the relative conservation of EBV sequences in Asia.

Although this study failed to identify statistically significant variants in the EBV DNA sequences of EBV-associated diseases patients from the perspective of disease subtypes and HLH incidence, our findings from other EBV-associated diseases provide indirect evidence of both internal homogeneity within EBV-associated diseases and heterogeneity across different conditions. BFLF1 variants, which are associated with an increased mortality risk, may provide reliable prognostic markers for the clinical outcome of EBV-associated diseases. Another limitation of our study is the neglection of host variations in critical immune surveillance genes (such as ITK, XIAP, CD27, and CTPS1) as well as HLA polymorphisms, which may also contribute to ethnic heterogeneities. Our future research will focus on expanding the sample size, incorporating patients with other EBV-related diseases and populations from diverse regions to further investigate DNA sequence polymorphisms. Additionally, we will employ multi-omics approaches such as transcriptomics and proteomics to conduct comprehensive analysis of EBV-associated diseases.

## Supplementary Information

Supplementary Material 1

## Data Availability

No datasets were generated or analysed during the current study.

## References

[1] Kanda T, Yajima M, Ikuta K. Epstein-Barr virus strain variation and cancer. Cancer Sci. 2019;110(4):1132–9. 10.1111/cas.13954. PMC6447851 30697862

[2] Münz C. Latency and lytic replication in Epstein-Barr virus-associated oncogenesis. Nat Rev Microbiol. 2019;17(11):691–700. 10.1038/s41579-019-0249-7. 31477887

[3] Quintanilla-Martinez L, Swerdlow SH, Tousseyn T, Barrionuevo C, Nakamura S, Jaffe ES. New concepts in EBV-associated B, T, and NK cell lymphoproliferative disorders. Virchows Arch. 2023;482(1):227–44. 10.1007/s00428-022-03414-4. PMC9852222 36216980

[4] Kimura H, Cohen JI. Chronic active Epstein-Barr virus disease: molecular pathogenesis, evolving concepts, and emerging therapies. Blood. 2026;147(14):1562–73. 10.1182/blood.2025032277. PMC13077486 41490284

[5] Janka GE. Hemophagocytic syndromes. Blood Rev. 2007;21(5):245–53. 10.1016/j.blre.2007.05.001. 17590250

[6] Walther LE, Ilgner J, Oehme A, Schmidt P, Sellhaus B, Gudziol H, Beleites E, Westhofen M. Die infektiöse Mononukleose [Infectious mononucleosis]. HNO. 2005;53(4):383–92. 10.1007/s00106-004-1210-1. quiz 393. German. 15657747

[7] Kawada JI, Ito Y, Ohshima K, Yamada M, Kataoka S, Muramatsu H, Sawada A, Wada T, Imadome KI, Arai A, Iwatsuki K, Ohga S, Kimura H. Committee for Guidelines for the Management of Chronic Active EBV Disease, Related Disorders (the MHLW Research Team in Japan). Updated guidelines for chronic active Epstein-Barr virus disease. Int J Hematol. 2023;118(5):568–76. 10.1007/s12185-023-03660-5. PMC10615970 37728704

[8] Miranda RN, Amador C, Chan JKC, Guitart J, Rech KL, Medeiros LJ, Naresh KN. WHO fifth edition classification project. Fifth Edition of the World Health Organization Classification of Tumors of the Hematopoietic and Lymphoid Tissues: Mature T-Cell, NK-Cell, and Stroma-Derived Neoplasms of Lymphoid Tissues. Mod Pathol. 2024;37(8):100512. 10.1016/j.modpat.2024.100512. 38734236

[9] Henter JI, Horne A, Aricó M, Egeler RM, Filipovich AH, Imashuku S, Ladisch S, McClain K, Webb D, Winiarski J, Janka G. HLH-2004: Diagnostic and therapeutic guidelines for hemophagocytic lymphohistiocytosis. Pediatr Blood Cancer. 2007;48(2):124–31. 10.1002/pbc.21039. 16937360

[10] Yuan L, Zhong L, Krummenacher C, Zhao Q, Zhang X. Epstein-Barr virus-mediated immune evasion in tumor promotion. Trends Immunol. 2025;46(5):386–402. 10.1016/j.it.2025.03.007. 40240193

[11] Latour S. Human Immune Responses to Epstein-Barr Virus Highlighted by Immunodeficiencies. Annu Rev Immunol. 2025;43(1):723–49. 10.1146/annurev-immunol-082323-035455. 40279309

[12] Murata T. Tegument proteins of Epstein-Barr virus: Diverse functions, complex networks, and oncogenesis. Tumour Virus Res. 2023;15:200260. 10.1016/j.tvr.2023.200260. PMC10200993 37169175

[13] Borozan I, Zapatka M, Frappier L, Ferretti V. Analysis of Epstein-Barr Virus Genomes and Expression Profiles in Gastric Adenocarcinoma. J Virol. 2018;92(2):e01239-17. doi: 10.1128/JVI.01239-17. Erratum in: J Virol. 2018;92(10):e00330-18. 10.1128/JVI.00330-18 PMC5752941 29093097

[14] Khine HT, Sato Y, Hamada M, Umeda M, Iizuka A, Son S, Arai H, Kojima Y, Watanabe T, Naruse A, Goto K, Ohshima K, Akutsu Y, Nakaguro M, Satou A, Kataoka H, Ito Y, Sawada A, Kato S, Kawada JI, Murata T, Okuno Y, Kimura H. Association of Epstein-Barr virus genomic alterations with human pathologies. Blood. 2025;146(13):1533–45. 10.1182/blood.2024028055 PMC12824656 40569273

[15] Murata T, Okuno Y, Sato Y, Watanabe T, Kimura H. Oncogenesis of CAEBV revealed: Intragenic deletions in the viral genome and leaky expression of lytic genes. Rev Med Virol. 2020;30(2):e2095. 10.1002/rmv.2095. 31845495

[16] Xu M, Yao Y, Chen H, Zhang S, Cao SM, Zhang Z, Luo B, Liu Z, Li Z, Xiang T, He G, Feng QS, Chen LZ, Guo X, Jia WH, Chen MY, Zhang X, Xie SH, Peng R, Chang ET, Pedergnana V, Feng L, Bei JX, Xu RH, Zeng MS, Ye W, Adami HO, Lin X, Zhai W, Zeng YX, Liu J. Genome sequencing analysis identifies Epstein-Barr virus subtypes associated with high risk of nasopharyngeal carcinoma. Nat Genet. 2019;51(7):1131–6. 10.1038/s41588-019-0436-5. PMC6610787 31209392

[17] Sugimoto A, Yamashita Y, Kanda T, Murata T, Tsurumi T. Epstein-Barr virus genome packaging factors accumulate in BMRF1-cores within viral replication compartments. PLoS ONE. 2019;14(9):e0222519. 10.1371/journal.pone.0222519. PMC6743757 31518362

[18] Pavlova S, Feederle R, Gärtner K, Fuchs W, Granzow H, Delecluse HJ. An Epstein-Barr virus mutant produces immunogenic defective particles devoid of viral DNA. J Virol. 2013;87(4):2011–22. 10.1128/JVI.02533-12. PMC3571473 23236073

[19] Khan G, Fitzmaurice C, Naghavi M, Ahmed LA. Global and regional incidence, mortality and disability-adjusted life-years for Epstein-Barr virus-attributable malignancies, 1990–2017. BMJ Open. 2020;10(8):e037505. 10.1136/bmjopen-2020-037505. PMC7462312 32868361

[20] Palser AL, Grayson NE, White RE, Corton C, Correia S, Ba Abdullah MM, Watson SJ, Cotten M, Arrand JR, Murray PG, Allday MJ, Rickinson AB, Young LS, Farrell PJ, Kellam P. Genome diversity of Epstein-Barr virus from multiple tumor types and normal infection. J Virol. 2015;89(10):5222–37. 10.1128/JVI.03614-14. PMC4442510 25787276

[21] Lin N, Ku W, Song Y, Zhu J, Lu Z. Genome-Wide Analysis of Epstein-Barr Virus Isolated from Extranodal NK/T-Cell Lymphoma, Nasal Type. Oncologist. 2019;24(9):e905–13. 10.1634/theoncologist.2017-0588. PMC6738302 30940744

[22] Li XR, Wu J, Yu SL, Zhou Z, Zhang WH, Shao LY, Jiang N, Yang QL. The impact of Epstein-Barr Virus genomic variations on clinical subtypes and prognosis in EBV-associated diseases. Url: https://data.4tu.nl/private_datasets/Mcui8_q05h_qSV71balx0le6Nl_RGPdGh7vTVfB9G_c

