# Supplementary material for: BFLF1 gene polymorphisms are associated with increased mortality in patients with EBV-associated diseases
Source: Hereditas. 2026 Jul 14;163:112. doi: 10.1186/s41065-026-00708-4 (PMC13663094; doi:10.1186/s41065-026-00708-4)
Supplement: Supplementary file 1 [file 41065_2026_708_MOESM1_ESM.docx]

**The impact of Epstein-Barr Virus genomic variations on clinical subtypes and prognosis in EBV-associated diseases**

**Authors：**

Xurong Li^1*^, Jing Wu^1*^，Shenglei Yu^1*^, Zhe Zhou^1^, Wenhong Zhang^1^，Lingyun Shao^1#^, Ning Jiang^1#^，Qingluan Yang^1#^

**Web-only Supplement**

Figure S1. Heatmap of quality control of raw data (A) through FastP (B) and Trimmomatic (C).

| Sample | Coverage | Sample | Coverage | Sample | Coverage |
| --- | --- | --- | --- | --- | --- |
| 108-yangyongming | 0.9984 | 97-zhuhuaiwang | 0.9951 | 71-bidan | 0.9758 |
| 104-liushuai | 0.9982 | 115-fanchangxi | 0.9933 | EB5 | 0.9621 |
| 53-huliangbin | 0.9977 | 10-LYF | 0.992 | EB3 | 0.9492 |
| 44-GJX | 0.9975 | 125-zhangyanfeng | 0.992 | EB9 | 0.4072 |
| 62-hejianhua | 0.9974 | EB27 | 0.9848 | EB7 | 0.225 |
| 112-wujian | 0.9973 | EB25 | 0.984 | 30-XFZ | 0.2235 |
| 100-luyitong | 0.9971 | EB17 | 0.9827 | EB20 | 0.0623 |
| 134-gaolijuan | 0.9968 | EB21 | 0.9827 | EB23 | 0.0611 |
| 102-yanshengwei | 0.9967 | EB24 | 0.9815 | EB2 | 0.0567 |
| 19-GJC | 0.9962 | EB1 | 0.9802 | EB10 | 0.0268 |
| 126-huangxiaoyi | 0.9956 | 72-wangtao | 0.9784 | EB8 | 0.0258 |
| 88-shanghengcui | 0.9952 | 132-panyulin | 0.9777 | 16-CHJ | 0.0083 |

Table S1. Coverage percentag of 36 patients.
